# Evolutionary Insight and Expression Pattern of WUSCHEL‐Related Homebox Genes of *Dendrobium huoshanense*

**DOI:** 10.1002/fsn3.70057

**Published:** 2025-02-23

**Authors:** Jing Wang, Yingyu Zhang, Yanshuang Ren, Muhammad Aamir Manzoor, Shanyong Yi, Cheng Song

**Affiliations:** ^1^ Department of Pharmacy The Affiliated Hospital of Nanjing University of Chinese Medicine, Jiangsu Province Hospital of Chinese Medicine Nanjing China; ^2^ Henan Key Laboratory of Rare Diseases, Endocrinology and Metabolism Center The First Affiliated Hospital, and College of Clinical Medicine of Henan University of Science and Technology Luoyang China; ^3^ Department of Plant Science, School of Agriculture and Biology Shanghai Jiao Tong University Shanghai China; ^4^ Anhui Dabieshan Academy of Traditional Chinese Medicine, Anhui Engineering Research Center for Eco‐Agriculture of Traditional Chinese Medicine, College of Biological and Pharmaceutical Engineering West Anhui University Lu'an China

**Keywords:** *Dendrobium huoshanense*, Genome‐wide analysis, homeodomain, phytohormone, WUSCHEL‐related homebox

## Abstract

The *WOX* (*WUSCHEL‐related homebox*) gene family is critical for plant growth, development, and the regulation of stress responses; however, the function of *Dendrobium huoshanense* WOX has not been extensively studied. Nine *WOX* genes have been identified in the *D. huoshanense* genome. These *WOX* genes were unequally distributed on five chromosomes, with four *WOX* genes occupying chr1. A phylogenetic tree of *D. huoshanense* and five other species was built based on the maximum likelihood method, suggesting that these WOX proteins could be grouped into three classical clades. Structural variation analysis of the three *Dendrobium* relatives revealed that *D. huoshanense* had more translocations and inversions with *D. nobile* than *D. chrysotoxum*. Intraspecific collinearity analysis of *D. huoshanense* based on the MCScanX revealed no large‐scale WGD and segmental duplications between these *WOX* genes. The Ka/Ks ratio and calculated timeline (MYA), indicate that all *DhWOX* genes were subject to purifying selection. Interspecies microsynteny analysis revealed that *D. huoshanense* shares more gene pairs with two closely related species, consistent with their genetic relationships. Gene structure analysis showed that *DhWOX1*, *DhWOX2*, *DhWOX6*, and *DhWOX8* contained 3′ or 5’ UTR. Conserved motif analysis revealed that these WOX contain conserved homeodomain domains and similar components. Multiple sequence alignments showed that the homeodomain near the C‐terminus has a typical helix–turn–helix structure and amino acid composition, while DhWOX3 uniquely contains WUS‐box motif. Protein–protein interaction analysis showed that DhWOX5 may be co‐expressed with DhWOX6 and DhWOX9. A large number of cis‐acting elements in the promoter regions associated with hormone signaling, vegetation development stages, and stress responses. Differential gene expression was analyzed after MeJA treatment. *DhWOX4* showed high expression at 2 and 4 h, whereas *DhWOX6* and *DhWOX7* showed dynamic fluctuations. *DhWOX8* showed consistently low expression, whereas DhWOX9 expression was highest at 2 and 4 h. *DhWOX1* and *DhWOX3* showed no detectable expression levels. The subcellular localization results indicated that *DhWOX2* and *DhWOX6* genes were expressed in the nucleus. These findings contribute to our understanding of the roles of these genes in *D. huoshanense*.

## Graphical abstract

Nine WOX genes were identified in *D. huoshanense*. The phylogenetic tree suggests they belong to three classical clades. All DhWOX genes are subject to purifying selection, with replication times dating back as early as 363 MYA. *D. huoshanense* has more gene pairs with its relative species, consistent with their genetic relationship. The cis‐regulatory elements in promoter regions are mainly associated with hormone signaling, vegetation development stages, and stress responses.

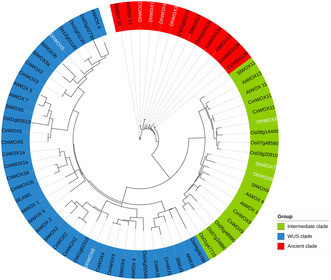

## 1 Introduction

The *WOX* (WUSCHEL‐related homeobox) family is a crucial group of transcription factors in plants (Wu et al. 2019). It belongs to the homeobox (HB) superfamily, a subset of the HB family. Members of this family have a single HD domain with a length of 60–66 amino acids (Lian et al. 2014). The WOX gene family is unique among HB families due to its single HD domain with a specific constituent sequence. This domain binds to DNA through a helix–turn–helix (HTH) structure, composed of two α‐helices and a shortened turn, both of which help the “HTH” structure to link directly with the DNA (Li et al. 2020a, 2020b). The WOX gene family plays a key role in plant development, primarily contributing to stem cell maintenance and differentiation across diverse plant family members, including monocots and dicots (Nakata et al. 2012). These genes operate through a shared DNA‐binding motif encoded within the homeodomain, which interacts with specific DNA sequences to regulate target genes essential for development (Segatto et al. 2016; Jadoon et al. 2024). Being tightly regulated, WOX genes show the highest activity just in narrow sections of plant tissues, as, for instance, in the shoot and root apical meristems. This controlled spatiotemporal regulation plays a role in different stages of plant development (Liu et al. 2014).

The WOX family is engaged in various activities. Some are involved in maintaining stem cell populations, whereas others are involved in controlling the differentiation of specific cell types during tissue development (Youngstrom et al. 2019). Although plant species differ from its peers, the structural and functional patterns of *WOX* genes have remained relatively consistent through evolutionary processes. This conservation demonstrates the primary significance of these genes for plant development in evolutionary biology (Zhu et al. 2016). The functions of *WOX* genes have been well studied in *A. thaliana* (Chen et al. 2023). The studies on genetically modified plants with altered *WOX* genes have provided further evidence of their functional aspects (Zhang et al. 2015; Li et al. 2020a, 2020b). Mutations in *WOX* genes can lead to observable defects in stem cell maintenance, organ formation, and overall plant morphology. Previous reports have shown that WOX genes support a broad range of life cycle functions, including embryonic development, embryonic polarization, meristematic maintenance, development of lateral organs, seeding, and regeneration of independent tissues and organs (He et al. 2022).

Orchidaceae is one of the largest and most widespread families within the realm of flowering plants, comprising over 28,000 species (Niu et al. 2020). Among these, Dendrobium, the second largest genus in the Orchidaceae family, has garnered considerable attention for its medicinal properties and ornamental appeal, captivating botanists and plant enthusiasts alike for centuries (Jiao and Luo 2021). Despite widespread documentation of *WOX* genes in model plants and major crops, little is known about the relationships and specific roles of *WOX* genes during flower development in Dendrobium species. In the recent years, sequencing of several *Dendrobium* species, including *D. chrysotoxum*, *D. huoshanense*, and *D. nobile*, has provided high‐quality chromosome‐level genome assemblies (Zhang et al. 2021; Han et al. 2020; Xu et al. 2022; Song et al. 2023). A bioinformatics approach was used to identify Dendrobium *WOX* genes by examining gene structure, motif composition, chromosomal localization, phylogenetic tree analysis, and segmental duplication analysis. Additionally, the expression patterns of *D. huoshanense* were investigated. These findings shed light on the biological functions of *DhWOX* genes and elucidate the molecular mechanisms underlying morphogenesis and stress responses in *D. huoshanense*.

## 2 Material and Methods

### 2.1 Retrieving Sequences From Databases

Amino acid sequences originating from *D. huoshanense* were extracted from the Medicinal Plants Multiomics Database (MPOD) at (http://applegbd.ynau.edu.cn/genome). In the MPOD database, the BLAST‐P (Basic Local Alignment Search Tool for Protein Sequences) tool (https://blast.ncbi.nlm.nih.gov/Blast.cgi) was used to extract WOX genes from *D. huoshanense* protein sequences using the hidden Markov model (PF00046) (Haider et al. 2023). A nonredundant protein database (NR) was used for sequence retrieval and alignment. The E‐value threshold was set to 1e‐5. Subsequently, the validity of the acquired amino acid sequences was confirmed through a comparative analysis against the Conserved Domain Database (CDD) (https://www.ncbi.nlm.nih.gov/Structure/cdd/wrpsb.cgi), InterPro (https://www.ebi.ac.uk/interpro/search/sequence/), and SMART (https://smart.embl.de/smart/set_mode.cgi) databases (Zhang et al. 2016).

### 2.2 Physical Properties, Subcellular Localization and Cis‐Acting Elements Analysis

The physical properties of the amino acid or protein data for the nine *WOX* proteins were gathered from two sources: Protparam (https://web.expasy.org/protparam/) and MPOD. MPOD provided details regarding the number and chromosomal locations as well as the orientation (sense or antisense) of the gene within specific regions, which were retrieved using GFF files. It also provides information on the CDS and peptide size. ProtParam software provided data such as the theoretical pI, molecular weight, Grand Average of Hydropathy (GRAVY), and stability index (SI) for these proteins. To predict subcellular localization, the Cell‐PLoc 2.0 (http://www.csbio.sjtu.edu.cn/bioinf/Cell‐PLoc‐2/) was utilized for predicting subcellular localization of WOX proteins. Additionally, 2000‐base pair upstream promoter regions were acquired from phytozomeV3 (https://phytozome‐next.jgi.doe.gov) (Hussain et al. 2024). The PlantCARE program (http://bioinformatics.psb.ugent.be/webtools/plantcare/html/) was used to predict the regulatory elements based on 5–20 base pairs of upstream sequences from the initial nucleotide in each case. The outputs were visualized as heat maps using TBtools (*v*.2.11) (Deng et al. 2024; Bülow and Hehl 2016; Ali et al. 2024).

### 2.3 Conserved Motif and Exon–Intron Analyses

The MEME‐Suite (*v*.5.5.7) program (https://meme‐suite.org/meme/tools/meme) was used to identify conserved motifs within amino acid sequences. It facilitates motif‐based analysis of DNA, RNA, and protein sequences, thus enabling the identification of motifs with arbitrary insertions and deletions, and offers tools for scanning sequences for motif matches. Subsequently, these sequences were submitted to the Conserved Domain Database (CDD) (https://www.ncbi.nlm.nih.gov/Structure/cdd/wrpsb.cgi), which utilized domains identified by NCBI as a reference database. It is a compilation of multiple sequence alignments and associated database search models representing protein domains conserved throughout molecular evolution. For the analysis of exon and intron distribution, the genomic and CDS sequences of the *DhWOX* gene family were processed using the Gene Structure Display Sever (GSDS) 2.0 web tool (http://gsds.cbi.pku.edu.cn/) (Barre et al. 2009; Hu et al. 2015). This was built for the visualization of annotated gene features and the production of high‐quality figures for publication, facilitating the display of exons/CDS coordinates alongside additional features.

### 2.4 Genomic Structural Variation Analysis

MUMmer 3 (https://sourceforge.net/projects/mummer/) was used to perform whole‐genome alignment between the two genomes. The reference genome was obtained from an accession link (https://ftp.cngb.org/pub/CNSA/data3/CNP0000830/CNS0251991/CNA0014590/). Two query genomes were obtained from the GWH database of NGDC with accession codes PRJNA725550 and PRJNA664445. GenomeSyn software (https://github.com/JM‐SONG/GenomeSyn) was downloaded and applied to visualize the genome syntenic blocks and structural variations between *D. huoshanense* and its two relatives.

### 2.5 Comparative Phylogenetic Analysis

To construct a phylogenetic tree, the amino acid sequences of the WOX proteins were aligned with those of *D. huoshanense*, *A. thaliana* , *Cucumis sativus* , *Solanum lycopersicum* , *Oryza sativa* , and *Cucumis melo* (Table S1) IQ‐TREE (*v*.1.6.6) software was used based on the maximum likelihood (ML) method. Bootstrapping was conducted with 1000 replications to enhance robustness. The best‐fit model for the ML tree was selected as VT + F + R4. Subsequently, the tree was visually adjusted using the iTOL program (https://itol.embl.de/) with default parameters, which enabled us to explore and annotate phylogenetic relationships (Rehman et al. 2022).

### 2.6 Selective Pressure, Microsynteny and Evolutionary Analysis

The microsynteny and evolution of *WOX* genes in *D. huoshanense* were investigated, focusing mainly on functional annotation and structural analysis through duplication and synteny blocks. The MUSCLE 5.3 (https://github.com/rcedgar/muscle) program was used to align the protein sequences, and the Ka/Ks ratio was determined using TBtools (*v*.2.11) with default settings, providing insights into the evolutionary pace of each gene pair. To ascertain the time of divergence of these genes, a formula incorporating T = Ks/2 and λ = 6.56 × 10^−9^ for monocots was used (Dos Reis et al. 2016). Gene duplications were detected using MCScanX (http://chibba.pgml.uga.edu/mcscan2/) with default settings (Wang et al. 2012). Microsynteny between *D. huoshanense* and other species was constructed according to their collinearity. Subsequently, TBtools (*v*.2.11) was used to create a dot plot showing the syntenic blocks between the paralogous genes in *D. huoshanense* (Luo et al. 2022).

### 2.7 Gene Structure and Protein–Protein Interaction Analysis

The GSDS 2.0 (http://gsds.cbi.pku.edu.cn/) web tool was selected to analyze the gene structure and intron–exon patterns. Additionally, the PlantCARE database (http://bioinformatics.psb.ugent.be/webtools/plantcare/html) was used to identify *cis*‐acting regulatory elements in the study (Zhang et al. 2023; Shafiq et al. 2024). The STRING database (https://string‐db.org) was used to explore the interactions among the *WOX* proteins. This database helped depict the relationships between the protein domains of the *WOX* proteins of *D. huoshanense* (Sami et al. 2024a, 2024b).

### 2.8 Expression Profile Analysis of *WOX* Genes

Transcriptome data from different periods were obtained from the GSA database of NGDC (accession CRA006607). Briefly, data from the control and MeJA‐treated groups at seven different time points were used to analyze the expression patterns of the *WOX* genes. We normalized the FPKM values, where values greater than 0 represented upregulation, and values less than 0 represented downregulation.

### 2.9 Subcellular Localization Analysis

The recombinant vector was created. The CDS sequences of the target genes *DhWOX2* and *DhWOX6* were obtained and compared with the plasmid map, in which the vector sequence should be placed. The single restriction enzyme site was analyzed with Primer Primer 5 software, and primers for *WOX2* and *WOX6* were generated. The target genes were amplified with the BioRun Magic PCR Mix Kit. The PCR product underwent agarose gel electrophoresis followed by gel recovery. The target gene and pAN580 vector were double‐digested with BsmB1 and Esp31 and thereafter combined, centrifuged, and incubated in a water bath at 37°C for 1 h, following the previous study provided (Gu et al. 2024). The constructed vector was linked to the target gene, mixed, and centrifuged immediately, then incubated at 37°C for 30 h. 5–10 μL of the ligation product was transferred to the competent *Escherichia coli* cells and incubated on ice for 30 min. Initially, submerge in water at 42°C for 90 s, followed by a 5‐min immersion in ice. 400 μL of LB liquid culture media was added and incubated on a shaker for 50 min. 200 μL of the supernatant was centrifuged and discarded, then resuspended and mixed with the remaining bacteria. 50 μL of that was transferred to inoculate the ampicillin‐resistant plate and incubated inverted at 37°C overnight. Plaques and bacterial suspensions were detected with the PCR method. Nicotiana benthamiana leaves were cultivated for 15 days, and 5–10 mL of enzymatic solution was prepared to immerse the tissue. The tissue was then kept at 24°C, and enzymatic hydrolysis was performed for 4 h. For the removal of the supernatant, this was filtered with a 40 μm filter and centrifuged. 200 μL of protoplast suspension was mixed with 20 μL of DNA (comprising 10 μL of target gene vector plasmid and 10 μL of marker plasmid). An equivalent amount of PEG 4000 solution was then added. The protoplasts were collected, and the supernatant was removed. 1 mL of W5 was added and performed 1–2 wash cycles. Subsequently, 1 mL of W5 solution was added and incubated in darkness at 25°C for 24 h. 100 μL of protoplasts are preserved and observed using a fluorescence microscope or laser confocal microscope.

## 3 Results

### 3.1 Physiochemical Properties of *WOX* Gene Family in *D. huoshanense*

Genome‐wide analysis was performed to determine *DhWOX* genes in the *D. huoshanense* genome. The HMM of the PF00046 domain was chosen as the query and a BLASTP search was performed on the *D. huoshanense* genome database hosted on MPOD. Nine *DhWOX* proteins have been identified. In the *DhWOX* gene family, four genes (*DhWOX2, DhWOX4, DhWOX5, and DhWOX7*) are oriented in the positive direction (+), whereas the remaining five genes (*DhWOX1, DhWOX3, DhWOX6, DhWOX8, and DhWOX9*) are oriented in the negative direction (−). Among the analyzed *DhWOX* genes, *DhWOX*8 demonstrated the highest instability with an index of 75.1, whereas *DhWOX*9 exhibited the lowest instability with an index of 62.1. The instability index ranges from 62.1 to 75.1 across the *DhWOX* family. Despite their variability, all *DhWOX* proteins exhibited instability, as their instability index values surpassed 40, implying potential dynamic behavior in their structural integrity. In terms of the coding sequence (CDS) length, *DhWOX*9 had the longest CDS with 951 amino acids, whereas *DhWOX*5 had the shortest CDS with 591 amino acids. Regarding peptide length, *DhWOX*7 has the longest peptide chain with 300 amino acids, whereas *DhWOX*5 has the shortest with 196 amino acids. Additionally, *DhWOX*9 exhibited the highest molecular weight (Mw) of 34273.88, whereas *DhWOX*5 presents the lowest Mw of 22141.97. Notably, *DhWOX*6 has the highest isoelectric point (pI) of 9.48, indicating a more basic pH environment for optimal activity. In contrast, *DhWOX*1 and *DhWOX*2 had the lowest pI values (5.26), suggesting activity under acidic conditions. The grand average of hydropathy (GRAVY) values suggests that all genes exhibit hydrophobic tendencies, with *DhWOX*2 having the least negative GRAVY score of −0.878 and *DhWOX*9 having the highest at −0.11. The prediction of subcellular localization showed that all DhWOX proteins were localized to the nucleus (Table 1).

**TABLE 1:** Physical properties of *WOX* genes discovered in *D. huoshanense* genome.

Gene ID	Rename	No.	Position	CDS	Pep	Mw	pI	SI	GRAVY	Subcellular localization
Dhu000017930	*DhWOX1*	Chr1	−	780	259	29924.5	5.26	66.63	−0.876	Nucleus
Dhu000017696	*DhWOX2*	Chr1	+	780	259	29910.47	5.26	65.97	−0.878	Nucleus
Dhu000014578	*DhWOX3*	Chr5	−	618	205	23751.03	6.33	68.5	−0.68	Nucleus
Dhu000025895	*DhWOX4*	Chr5	+	684	227	26353.92	6.2	66.47	−0.717	Nucleus
Dhu000026619	*DhWOX5*	Chr12	+	591	196	22141.97	9.11	65.29	−0.758	Nucleus
Dhu000022496	*DhWOX6*	Chr1	−	822	273	29665.28	9.48	70.79	−0.472	Nucleus
Dhu000007886	*DhWOX7*	Chr1	+	903	300	32638.97	6.31	63.71	−0.17	Nucleus
Dhu000027195	*DhWOX8*	Chr8	−	690	229	25903.44	7	75.1	−0.691	Nucleus
Dhu000021560	*DhWOX9*	Chr19	+	951	316	34273.88	7.66	62.1	−0.11	Nucleus

### 3.2 Chromosome Mapping Analysis of *DhWOX* Genes

**FIGURE 1:**
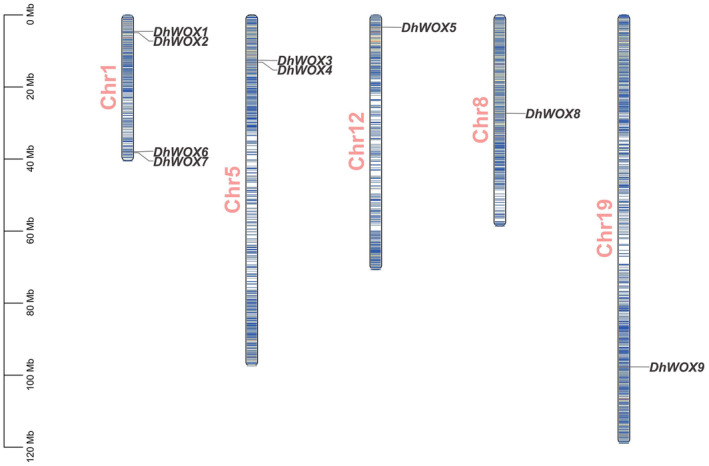
Chromosomal mapping of the DhWOX genes within the *D. humoshanense* genome.

The results of the chromosome mapping analysis shed light on the genome of the *DhWOX* genes. Among the mapped genes, *DhWOX*1, which is located on chromosome 1, had a minimum total length of 40,689,229 base pairs. In contrast, *DhWOX*9, which is located on chromosome 19, had the highest total length, covering 119,107,033 base pairs. This wide variability in total length emphasizes the extent of genomic size variation among the *DhWOX* genes. Furthermore, the distribution of genes across chromosomes revealed variations: multiple genes were located on specific chromosomes, whereas others were distributed more separately. *DhWOX1*, *DhWOX2*, *DhWOX6*, and *DhWOX7* are located on chromosome 1, whereas *DhWOX3* and *DhWOX4* are on chromosome 2. *DhWOX8* was anchored to chromosome 8, whereas *DhWOX5* was anchored to chromosome 12. DhWOX9 is the only gene on chromosome 19 (Figure 1).

### 3.3 The Phylogenetic Analysis of the WOX Family

**FIGURE 2:**
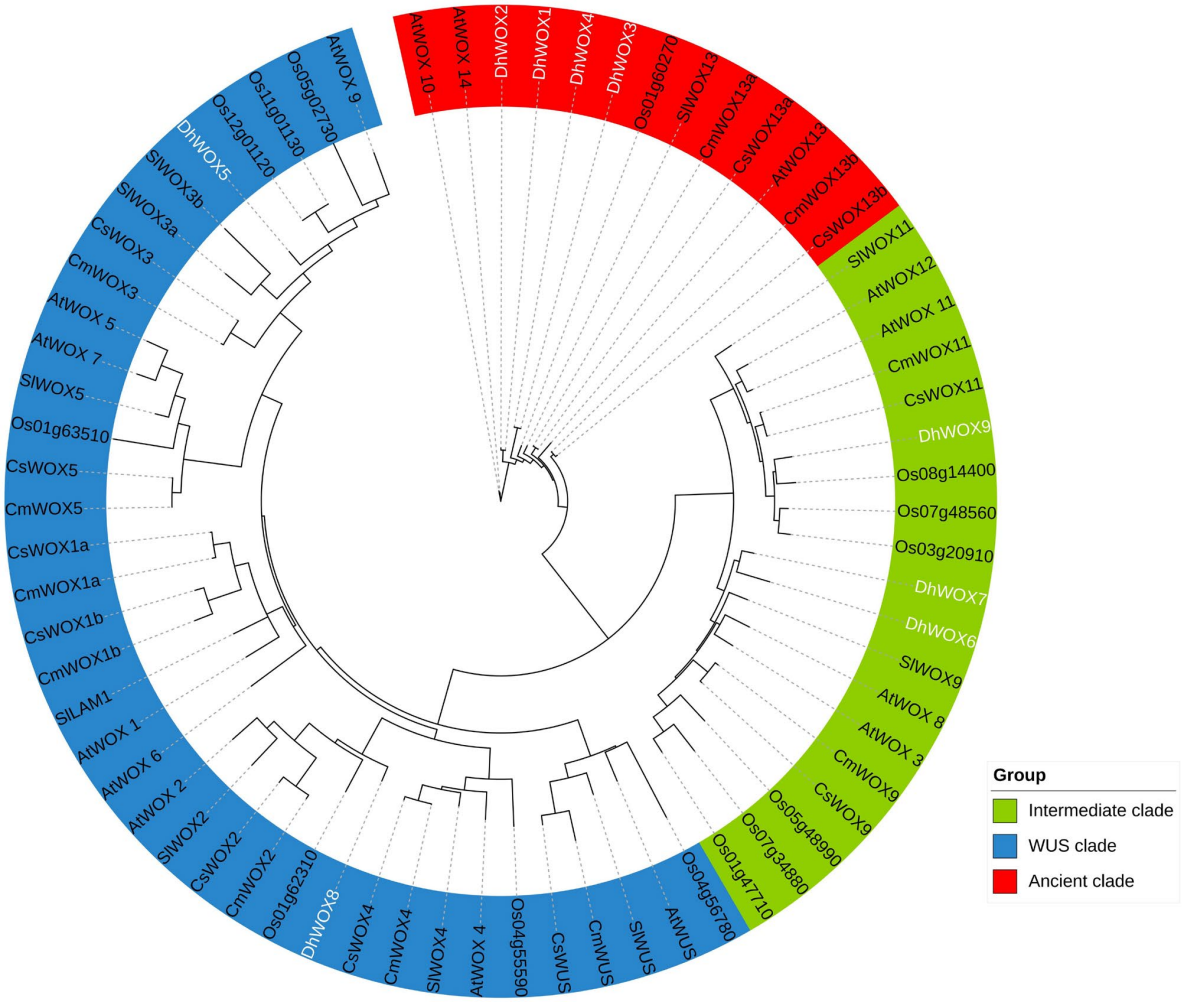
Phylogenetic analysis of *WOX* genes constructed from five plant species. The genetic tree separated the genes into three different clusters referred to as ancient clade, intermediate clade, and WUS clade.

For the phylogenetic analysis of WOX proteins, we used protein sequences from six plant species: *D. humoshanense*, *A. thaliana* , *C. sativus* , *S. lycopersicum* , *O. sativa* , and *C. melo* . The phylogenetic tree separated the genes into three different clusters, referred to as intermediate, WUS, and ancient clades. The results showed that the ancient clade contained four genes, from DhWOX1 to DhWOX4, followed by an intermediate clade containing DhWOX6, DhWOX7, and DhWOX9. The WUS clade contained only DhWOX5 and DhWOX8. DhWOX and some homologous genes in rice clustered better into one branch (Figure 2). The resulting phylogenetic tree revealed the genetic relationships and evolution of the WOX genes among the representative plant species.

### 3.4 Analysis of Genetic Structural Variation Among Dendrobium Species

**FIGURE 3:**
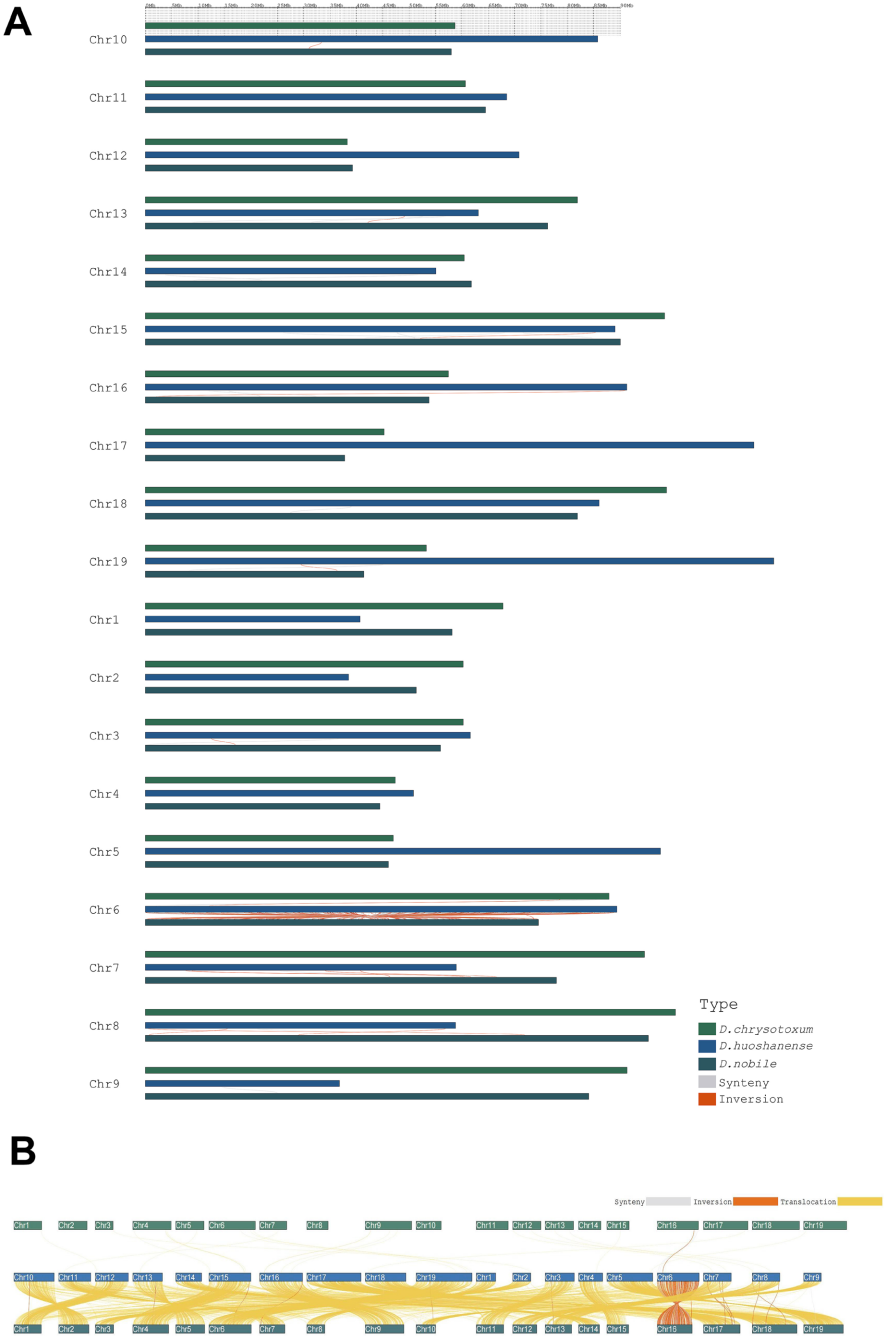
Structural variation in the genomes of *D. huoshanense* and other Dendrobium species. (A) Distribution of synteny and inversion among 19 pairs of chromosomes. (B) Synteny, translocation, and inversion distribution among all chromosomes.

In determine the structural variation events between *D. huoshanense* and its close relatives, we used Mummer software for whole‐genome comparisons. GenomeSyn analysis results showed that *D. huoshanense* had certain collinear modules with *D. chrysotoxum* and *D. nobile*, but there were more inversion events between them, mainly on chromosome 6, followed by chromosomes 7 and 8 (Figure 3A). Additionally, there is a translocation between almost all the chromosomes of *D. huoshanense* and *D. nobile*, which plays a crucial for genome evolution in the genus Dendrobium. A smaller number of translocations were observed between *D. huoshanense* and *D. chrysotoxum*, which were mainly distributed on chromosomes 15, 16, and 19 (Figure 3B).

### 3.5 Selection Pressure Analysis Among *DhWOX* Genes

**FIGURE 4:**
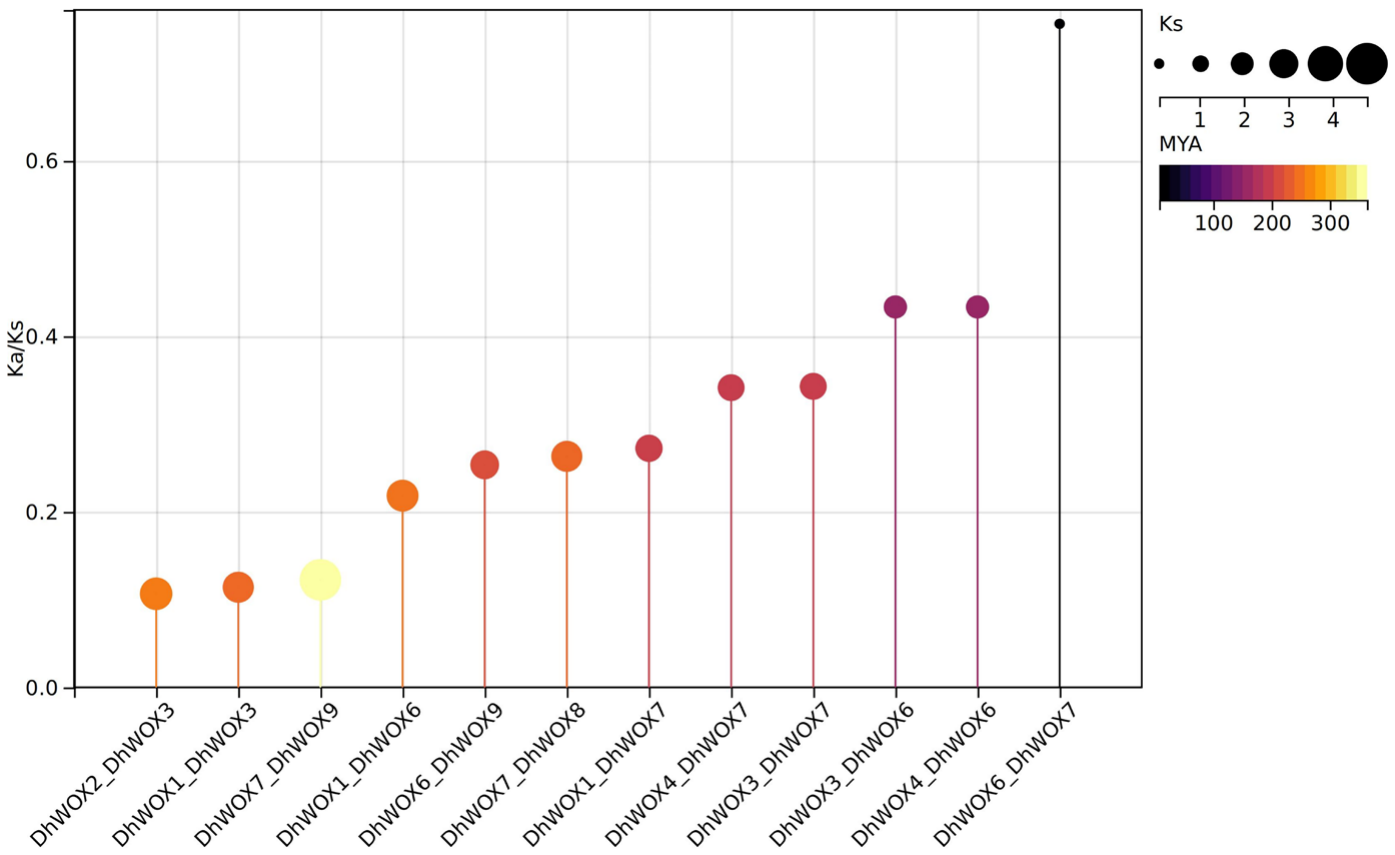
The expression Ka/Ks represents the ratio of mutations involving synonymous substitutions (Ks) to mutations involving non‐synonymous substitutions (Ka). Gene duplication over selection and evolutionary pressure to paralogous pairings of *D. huoshanense* genes were calculated based on Ks and Ka values. Gene replication time span is MYA.

Twelve gene pairs were compared in the Ka/Ks analysis, which indicated different levels per nonsynonymous site (Ka), and therefore offered information about the effects exerted by selective forces on these genes (Table S2). The DhWOX6_DhWOX7 pair has the lowest Ka value of 0.073, indicating that these two genes have a low number of amino acid differences. Similarly, the largest Ka ratio (0.886) was observed in the DhWOX3_DhWOX7 family, indicating a greater degree of amino acid divergence between these two genes. The Ka values of these gene sequences provide important data on the acceleration of evolutionary and functional changes within the *DhWOX* gene family. In contrast, the Ks test helped us understand the evolutionary divergence between the gene pairs. Notably, the DhWOX6_DhWOX7 pair had the lowest Ks value (0.097), indicating a corresponding divergence time of approximately 7.36 million years ago (MYA). However, the DhWOX7_DhWOX9 group had the highest Ks value of 4.77, confirming that this group diverged about 363.59 MYA. These Ks values differentiated and estimated the divergence times of *DhWOX* gene pairs with varying degrees of genetic evolution. The Ka/Ks ratio, indicating selective pressure, and *DhWOX* gene pairs were found to have notable differences. DhWOX2_DhWOX3 exhibited the smallest Ka/Ks ratio of 0.107, implying that purifying selection, which is essentially a conserved protein function, predominantly acts on these genes. In contrast, the DhWOX6_DhWOX7 pair showed the highest Ka/K ratio of 0.756. This suggests that positive selection might be sufficient to induce amino acid changes in these genes (Figure 4).

### 3.6 Gene Duplication and Synteny Analysis of *DhWOX* Genes

**FIGURE 5:**
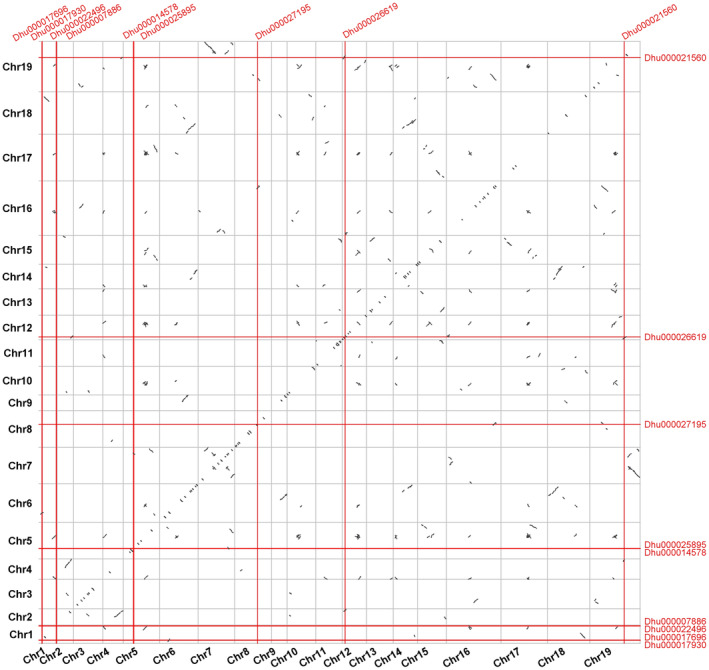
Whole‐genome duplication analysis based on dotplot mapping. The red intersection line is the potential duplication events.

**FIGURE 6:**
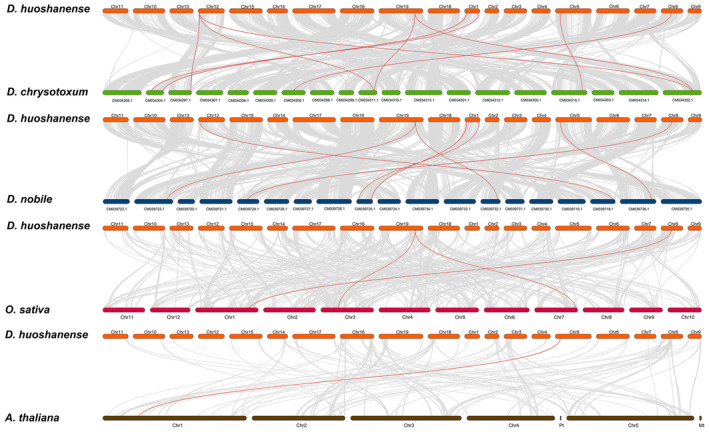
Microsynteny analysis of *D. huoshanense* and other species. Red connecting lines represent collinear gene pairs.

We used MCScanX collinearity analysis and constructed a dot plot to determine how the *WOX* genes were obtained through gene duplication. The intersection points in the figure do not fall on the solid black line in the background, indicating that there is no whole‐genome or segmental duplication between the *WOX* genes. Therefore, we inferred that it could originate from a proximal and dispersed transposition or insertion (Figure 5). Collinearity between species showed that there were more synteny blocks among *D. huoshanense*, *D. nobile*, and *D. chrysotoxum*, suggesting that they were closely related. There were very few collinear gene pairs between *D. huoshanense*, *O. sativa* , and *A. thaliana* , with only three and one pair, respectively (Figure 6). These results indicated that the origin of *DhWOX* genes was not generated by large‐scale gene duplication.

### 3.7 Gene Structure and Conserved Motif Analysis of DhWOX Genes

**FIGURE 7:**
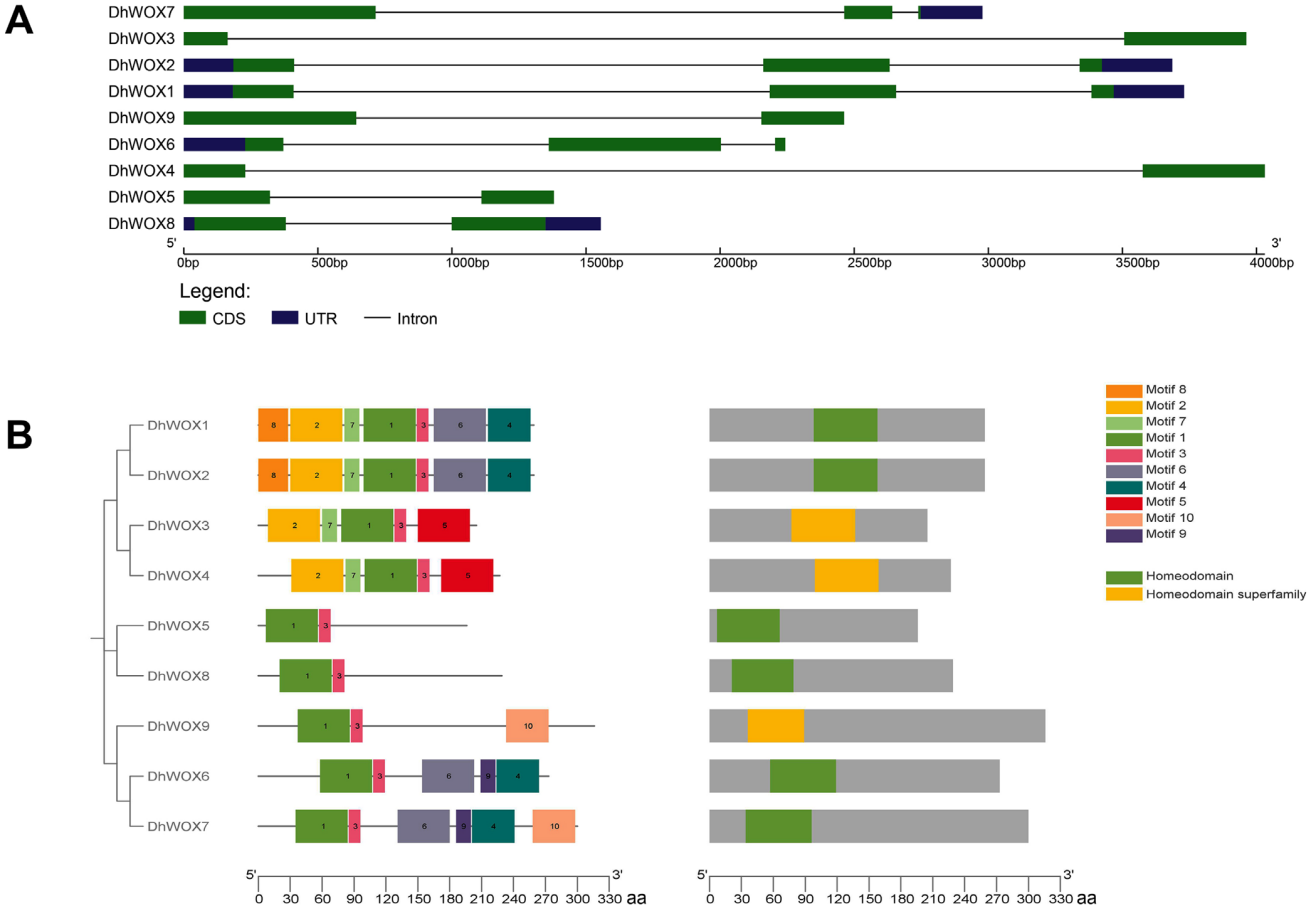
Gene structure and conserved motif analysis. (A) Intron–exon architecture patterns a among *DhWOX* genes, highlighting unique patterns, which make them stand out from the other ones. (B) Conserved domain analysis identifies that all 9 *DhWOX* proteins contain a conserved homeodomain domain, indicating their multiple regulatory role in plant growth and development. The distribution of 10 motifs across the 9 members of the *DhWOX* genes protein family.

During the exon–intron distribution analysis, *DhWOX1* and *DhWOX2* displayed very similar patterns and comprised two introns and three exons, respectively. Similarly, *DhWOX6* maintained the pattern of two introns and three exons. *DhWOX3*, *DhWOX4*, *DhWOX5*, *DhWOX8*, and *DhWOX9* had more basic structures with one intron and two exons. *DhWOX7* contains two introns and two exons. The data showed a thorough intron–exon pattern correlation between different *DhWOX* genes as well as unique patterns, which made them stand out from the others (Figure 7A). Domain analysis showed that the homeodomain region was conserved among all *DhWOX* genes. Specifically, DhWOX1, DhWOX2, DhWOX5, DhWOX6, DhWOX7, and DhWOX8 exhibited a specific hit for the homeodomain (PSSM‐ID: 459649), which was distributed in superfamily CL47488 according to accession number pfam00046. The fact that they all contain a homeodomain suggests that their functional significance may be similar among members of the group. DhWOX3, DhWOX4, and DhWOX9 are within the homeodomain superfamily (cl47488), indicating that homeodomains are synonymous with these genes (Figure 7B). Using motif analysis, we identified motif patterns unique to DhWOX genes. DhWOX1 and DhWOX2 had the same motifs located at positions 1, 2, 3, 4, 6, 7, and 8. Similarly, DhWOX6 and DhWOX7 displayed motifs at positions 1, 3, 4, 6, and 9. DhWOX9 displayed motifs at sites 1, 3, and 10. DhWOX3 and DhWOX4 have similar motifs at positions 1, 2, 3, and 7, whereas DhWOX4 has a motif at position 5. DhWOX5 was identified at positions 1 and 3. DhWOX8 contains motifs at positions 1 and 3. Among these motifs, motif 1 was the most conserved and was present in all the DhWOX genes examined (Figure 7B).

### 3.8 Multiple Sequence Alignment of DhWOX Proteins

**FIGURE 8:**
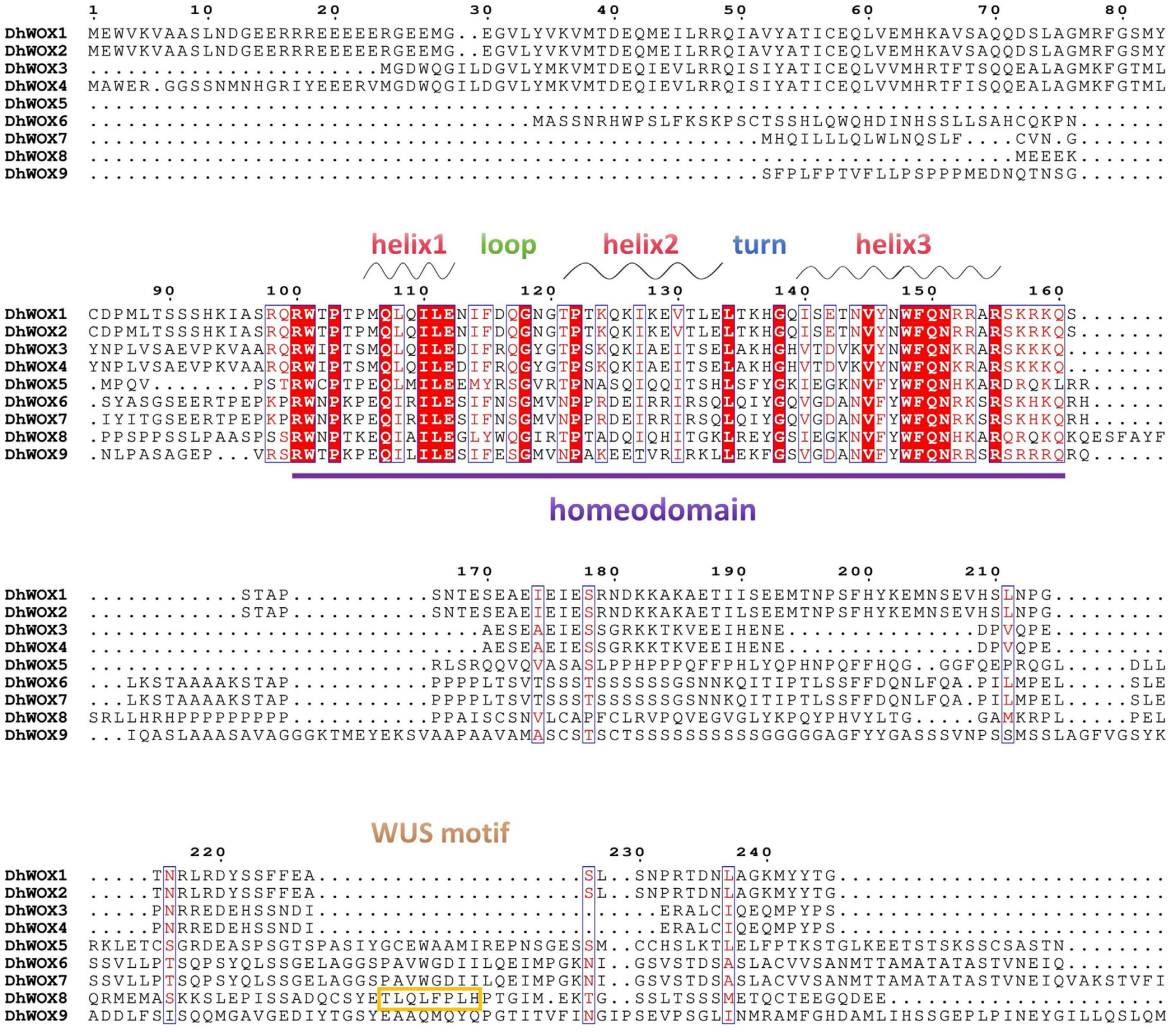
Amino acid composition and conserved domains of DhWOX proteins.

The DhWOX protein has a typical homeodomain, which is a highly conserved helix–loop–helix–turn–helix found in all WOX families, including two conserved motifs: R‐W‐X‐P‐X‐X‐X‐Q‐X‐X‐I‐L‐E and V‐Y/F‐X‐W‐F‐Q‐N‐X‐R/K‐X‐R. A T‐L‐Q‐L‐F‐P‐L‐H octapeptide is present at the C‐terminus of DhWOX8, which is the original WUS‐box motif. We did not detect an ERF‐associated amphiphilic repression (EAR) motif in DhWOX (Figure 8).

### 3.9 Protein–Protein Interaction

**FIGURE 9:**
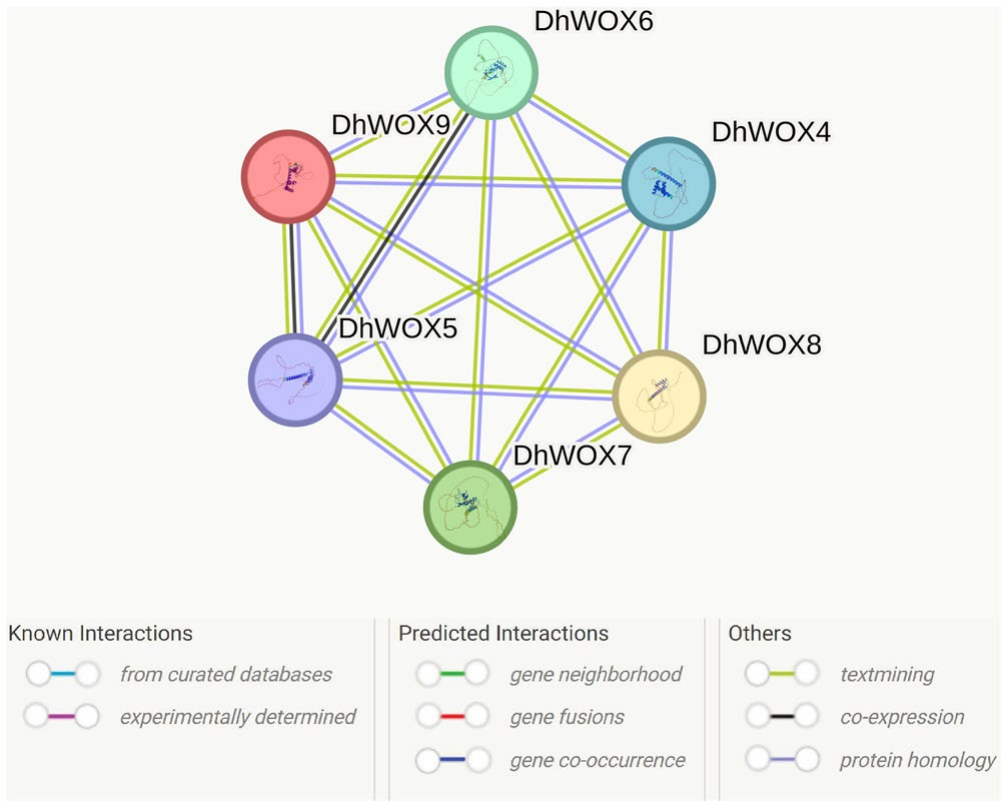
Protein–protein interaction indicated that six WOX proteins exhibited strong connectivity, suggesting their collective involvement in gene expression processes.

PPI analysis revealed a highly connected and interactive network containing six genes: DhWOX4, DhWOX5, DhWOX6, DhWOX7, DhWOX8, and DhWOX9. DhWOX4 interacts with (DhWOX5, DhWOX6, DhWOX7, DhWOX8, DhWOX9), DhWOX5 (DhWOX4, DhWOX6, DhWOX7, DhWOX8, DhWOX9), DhWOX6 (DhWOX4, DhWOX5, DhWOX7, DhWOX8, DhWOX9), DhWOX7 (DhWOX4, DhWOX5, DhWOX6, DhWOX8, DhWOX9), DhWOX8 (DhWOX4, DhWOX5, DhWOX6, DhWOX7, DhWOX9), and DhWOX9 with (DhWOX4, DhWOX5, DhWOX6, DhWOX7, DhWOX8). These interactions totaled 15 and indicated the number of edges between nodes. The average degree of connectivity for each node was approximately 5, indicating the broad depth of the network (Figure 9). The average local clustering coefficient, which serves as an indicator of the propensity of nodes to cluster, was measured as 1, implying a high level of clustering in the network. The expected number of edges in the network was 1, suggesting that the observed interactions were significantly enriched beyond random chance (*p* < 1.0 e^−16^). To ensure that the interactions are trustworthy, a minimum confidence score of 0.150, or low confidence, was selected.

### 3.10 Functional Classification of Cis‐Acting Element of *DhWOX* Genes

**FIGURE 10:**
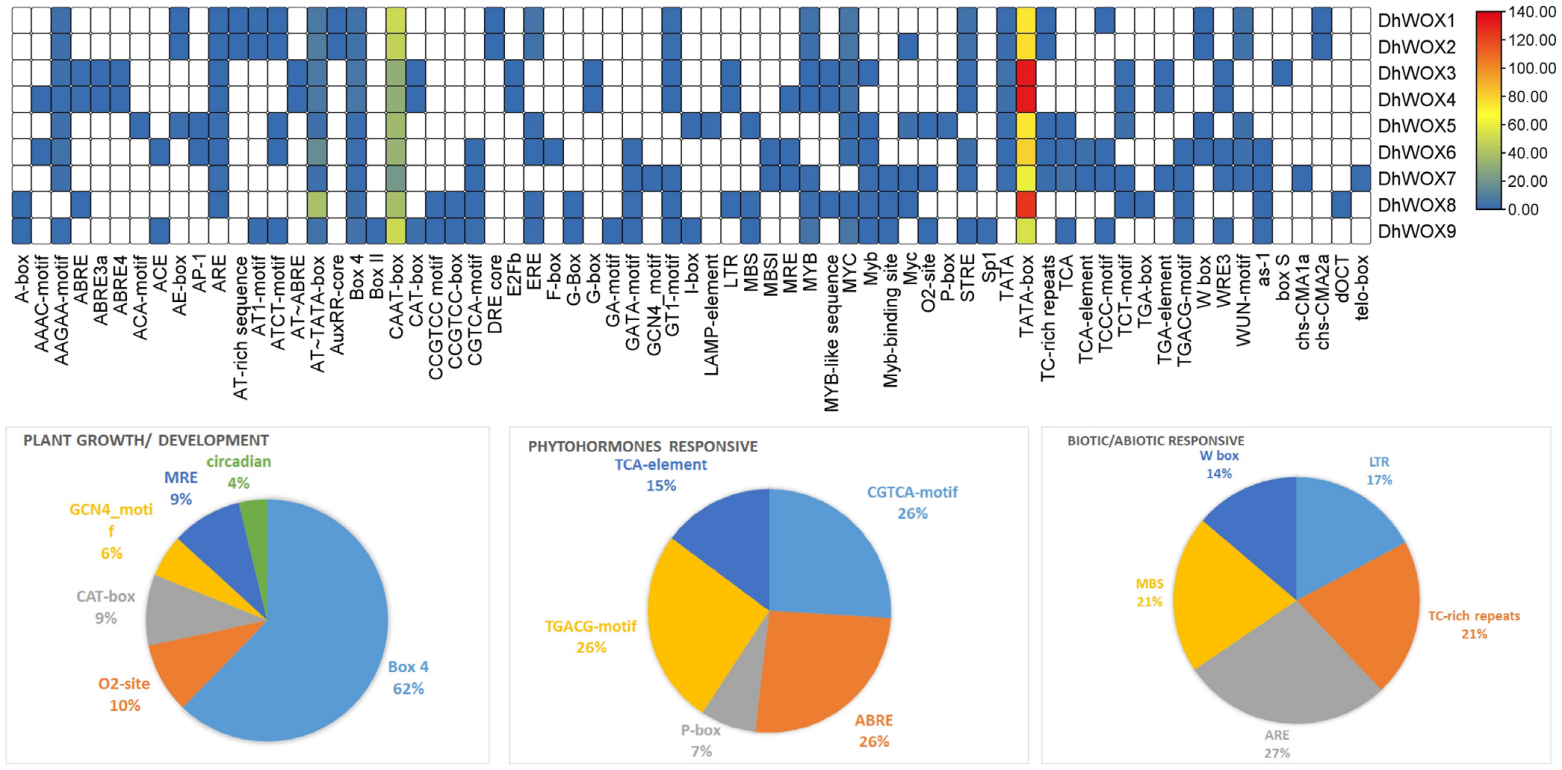
Cis‐acting element analysis of the *DhWOX* gene and the magnitude of each function. The intensity of the biochemical and physiological processes occurring in plants is ranked from red (highest) to blue (lowest).

Sixty‐nine cis‐acting element obtained from the genomic sequences were selected for further study (Table S3). These elements were classified into distinct groups based on their functions and associations. Some of the cis‐elements identified were relevant to light responsiveness. These included the G‐Box, AE‐Box, Myb‐binding site, Myb, Myc, and Myc‐binding sites, suggesting that they may play a role in the regulation of genes. Development‐related cis‐elements, such as E2Fb, ACE, and AP‐1, are involved in developmental processes. Stress‐responsive elements, such as the DRE core, MYB‐like sequence, and TC‐rich repeats, play roles in the response to various environmental stresses. Promoter‐related cis‐elements such as Box II, ACA‐motif, GATA‐motif, and CAT‐box are associated with promoter activity and transcriptional regulation. Notably, the TATA‐box motif emerged as the most prevalent motif, appearing 810 times out of 1606 instances, representing 50.44% of total occurrences. The core promoter element is essential for transcriptional initiation. Similarly, the CAAT‐box motif was highly abundant, with 337 occurrences, accounting for 20.98% of the total number. Both motifs play crucial roles in regulating gene expression by facilitating the assembly of the transcriptional machinery. The other motifs had fewer notable occurrences. For instance, the MYC motif appeared 30 times, contributing 1.87% of the total occurrences, whereas the MYB motif appeared 18 times, representing 1.12%. The cis‐acting elements in these regions modulate the expression of genes responsible for cell division, DNA synthesis, and stress responses. Medium frequencies were also discovered in addition to the AAGAA motif and Box 4, which had 23 and 33 recurrences, respectively. Such motifs may act as regulators of specific pathways, but this requires further research. In addition, the other cis‐elements, STRE, ERE, and TATA, each represented ~1% equally (Figure 10).

### 3.11 Expression Profile Analysis of *DhWOX* Genes

**FIGURE 11:**
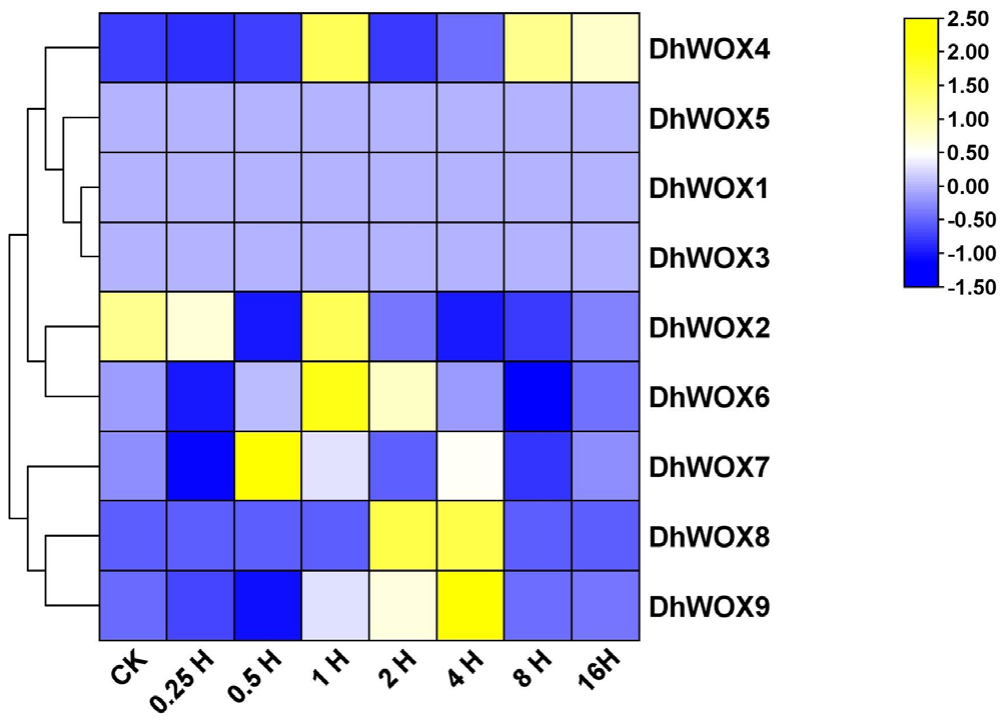
Expression patterns of *DhWOX* genes at different stages induced by MeJA. MeJA was applied on *D. huoshanense* and the expression levels of various genes over different time intervals ranging from 0.25 to 16 h were observed.

We assessed the gene expression levels, measured in fragments per kilobase of transcript per million mapped reads (FPKM), at different time points following MeJA treatment during our RNA sequencing analysis. MeJA was applied for the first time at 0.25 h, followed by subsequent applications at 0.5 h, 1 h, 2 h, 4 h, 8 h, and 16 h. CK represents the baseline expression levels before any treatment. For *DhWOX2*, there was a noticeable fluctuation in expression levels across the time points, with a relatively high expression of 76.023 observed at 0.25 h, which is still less than the control group's expression of 82.753, followed by a decrease at subsequent time points and a subsequent increase at 16 h. Conversely, *DhWOX4* displayed low expression initially, followed by a marked increase at 2 h and 4 h time points, and then a slight decrease at 8 h and 16 h. *DhWOX6* and *DhWOX7* exhibited varied expression patterns, with fluctuations observed across different time points. *DhWOX8* showed consistently low expression across all time points, whereas *DhWOX9* displayed a fluctuating pattern with peaks observed at 2 and 4 h. Notably, *DhWOX1* and *DhWOX3* did not show detectable expression levels over time (Figure 11).

### 3.12 Subcellular Localization of the *DhWOX2* and *DhWOX6* Genes

**FIGURE 12:**
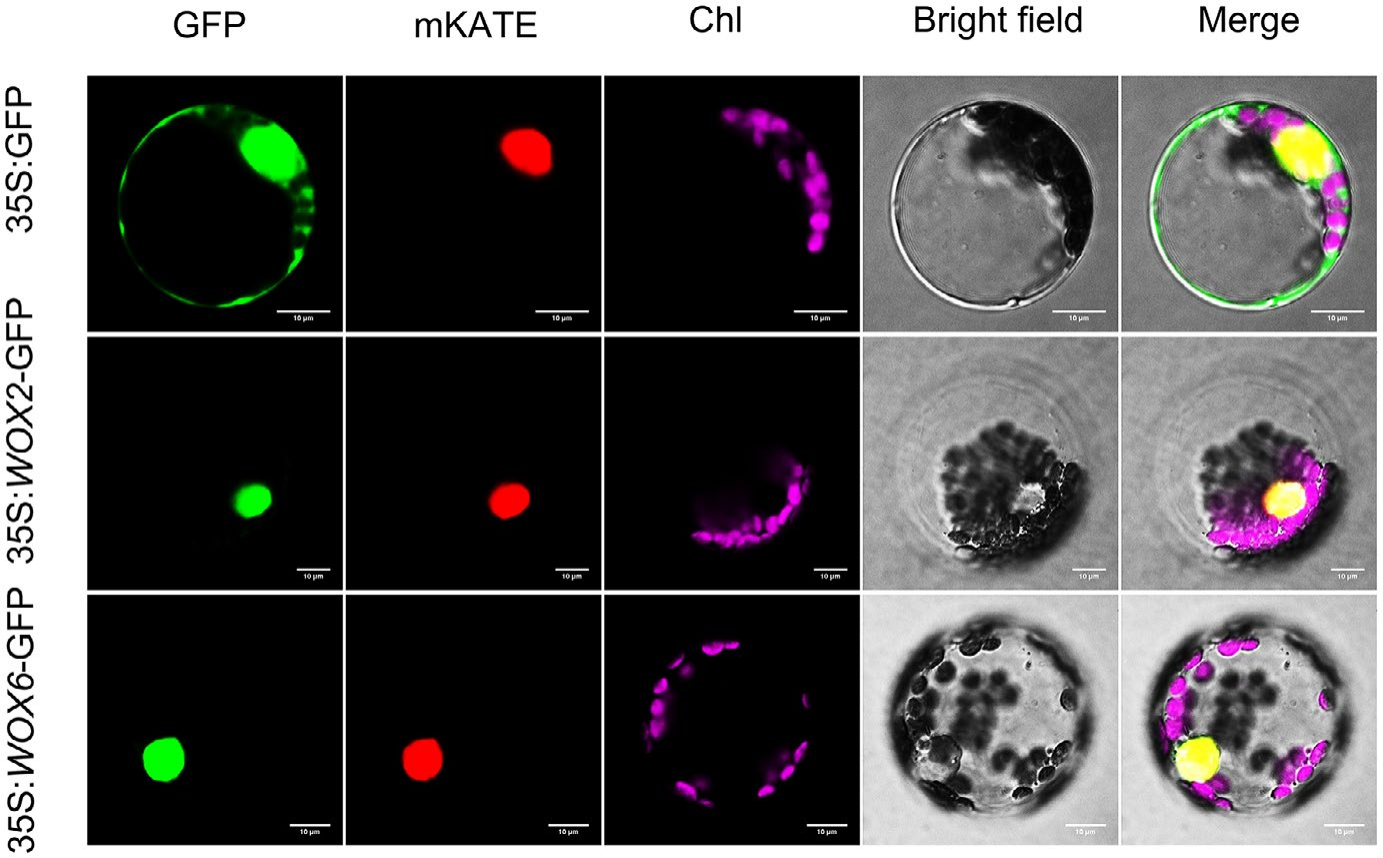
Subcellular localization of *DhWOX2* and *DhWOX6*. 35S:GFP: empty control. GFP: green fluorescence channel. mKATE: the nuclear marker red fluorescence channel. Chl: chloroplast channel. Bright field: bright field view. Merge: overlay image.

The fusion protein was developed by recombining the coding sequences of the *DhWOX2* and *DhWOX6* genes with the GFP gene, co‐localized with the red fluorescent protein (mKATE) as a nucleus localization marker, and transiently expressed in tobacco protoplasts. The protoplasts with the GFP control had green fluorescence in both the cytoplasm and the nucleus. On the other hand, the protoplasts that were infused with *WOX2*‐GFP and *WOX6*‐GFP only had fluorescence signals in the nucleus. The overlap of the red fluorescent label in the nucleus with the green fluorescence of GFP, which was proven that DhWOX2 and DhWOX6 were localized in the nucleus (Figure 12).

## 4 Discussion

The *WOX* gene family plays an important role in plant development and stress responses. Studies have shown its participation in abiotic stress responses such as drought, salt, and cold stress. The expression of *WOX* genes has been observed in response to abiotic stress, particularly in tomatoes (Li et al. 2021; Costanzo et al. 2014; Breuninger et al. 2008). Treatments with hormones such as gibberellin, abscisic acid, indole‐3‐acetic acid, jasmonic acid, and salicylic acid can also induce the expression of *WOX* genes (Sami et al. 2024a, 2024b). Further investigation is required to determine the interaction of *WOX* genes interact with MeJA. Under various stress conditions, the application of MeJA to *D. huoshanense* can provide plant protection. WOX genes are key regulators of different developmental processes in various plant species (Breuninger et al. 2008). However, the specific contributions of *DhWOX* genes to development, growth, and evolutionary adaptation strategies remain unclear. The whole‐genome sequence of *D. huoshanense* provides clues to studying the *DhWOX* family in a systematic manner. In this study, we identified nine *DhWOX* genes in *D. huoshanense*, and the results of structural and phylogenetic analyses confirmed the reliability of our findings. Arabidopsis contains 14 *AtWOX* genes. This may be due to the faster evolution of Arabidopsis than that of *D. huoshanense* (Dolzblasz et al. 2016; Hao et al. 2019). To identify the differences between proteins in the same clade, we investigated the physicochemical properties of DhWOX genes in the *D. huoshanense* genome. All identified *D. huoshanense* proteins had negative GRAVY values, indicating that they preferred to interact with water and had net electrical charges at different pH levels (Cheng et al. 2022). The instability index revealed that all the nine proteins were unstable. Subcellular localization analysis revealed that distribution of DhWOX proteins are primarily distributed throughout the nucleus, suggesting that DhWOX proteins play an important role in these parts of the cell (Li et al. 2019).

Previous studies have emphasized the significance of exon–intron organization in the evolution of gene families (Zhang et al. 2024). Our analysis revealed that the members within the same population and clade shared similar exon, intron, and motif distributions, which is consistent with the structure of phylogenetic trees (Khan et al. 2018; Song et al. 2024). All DhWOX proteins contained exons and introns. Notably, among these motifs, motif 1 was the most conserved and present in all DhWOX genes examined. Domain analysis showed that the homeodomain region was conserved and present in all DhWOXs, indicating its fundamental and evolutionary significance in this gene family. Because these conserved motifs are part of the conserved domain homeodomain, we believe that the intron–exon and special functions of these motifs can be explained by referring to the results of the protein multiple sequence alignment. For example, it was observed in the gene structure that *DhWOX1*, *DhWOX2*, and *DhWOX8* possess complete 3′ and 5' UTRs, indicating that their expression may be regulated normally and have reasonable functions (Song et al. 2021). *DhWOX6* only contains 5' UTR, and *DhWOX7* only contains 3' UTR. This difference suggests that they may have different expression levels, whether as transcription factors or main effector genes. Phylogenetic analyses can assist in understanding functional genomics by revealing similarities among subgroups (Yang et al. 2017). In the present study, WOX proteins with complete domain sequences were classified into two subfamilies based on their sequence structures and phylogenetic relationships. Within this subgroup, nine DhWOX proteins were identified, indicating potential functional similarities to AtWOX proteins. Protein–protein interaction analysis indicated that six proteins exhibited strong connectivity, suggesting their collective involvement in gene expression processes (Li et al. 2021). Analyzing genomes across species offers insights into gene evolution and organization, facilitating the transfer of genomic data from well‐studied to less‐explored taxa (Jain et al. 2008). In this study, we identified 12 pairs of paralogous genes in the genome that likely originated from gene duplications. Duplication analysis revealed varying divergence times estimation revealed varying divergence times, with lower MYA values indicating more recent divergence for pairs such as *DhWOX 6_DhWOX 7*, with higher MYA values indicate ancient divergence for pairs such as *DhWOX 7_DhWOX 9*. These duplications yield valuable insights into the expansion of gene families, a common phenomenon in plants driven by tandem and segmental duplications (Du et al. 2023).

Cis‐acting regulatory elements in gene promoter regions play major roles in regulating gene expression and transcriptional activity (Shafique Khan et al. 2021). The analysis of cis‐regulatory elements revealed that a significant proportion of the elements (49.06%, 53 of 109) responded to light growth and development, featuring motifs such as Box 4, MRE, and G‐box. The second largest group (27%, 29 of 109) was associated with abiotic and biotic stress and contained motifs such as LTR, TC‐rich repeats, ARE, MBS, and the W box. The third largest group, comprising 25% of the total, was made up of plant hormones. These had patterns such as the CGTCA‐motif and TGACG‐motif for the MeJA response; TCA‐element for the SA response; GARE‐motif, TATC‐box, and P‐box for the GA response; ABRE for the ABA response; and TGA‐element for the auxin response. These findings suggest potential roles for the *DhWOX* genes in regulating the responses to various abiotic stresses and growth (Rathour et al. 2020). Transcriptome analysis of *DhWOX* genes in the MeJA treatment revealed distinct expression patterns. The expression of *DhWOX2* changed over time and reached its highest level at 0.25 h, but was lower than that in the control group. In the case of *DhWOX4*, it had a low expression throughout 0 h, then a significant increase at 2 h and 4 h, before a slight decrease at 8 h and 16 h. *DhWOX6* and *DhWOX7* were expressed with variations, indicating a trend that was observed at different times. *DhWOX8* showed consistently low expression at all time points, whereas *DhWOX9* showed a fluctuating expression pattern, with peaks at 2 and 4 h. This suggests that *DhWOX6*, *DhWOX7*, and *DhWOX9* function according to specific MeJA concentrations and optimal timeframes. In addition, *DhWOX1* and *DhWOX3* showed low expression levels throughout the time interval, which indicated a lack of response to MeJA under the conditions applied (Li et al. 2024; Cao et al. 2019; Hakata et al. 2017).

WOX genes show spatiotemporal and tissue‐specific expression, reflecting their roles at different developmental levels (Pan et al. 2024). Our study demonstrated the pivotal role of some *DhWOX* genes in the response of *D. huoshanense* to MeJA. The results of the expression profile revealed that *DhWOX2* was the earliest MeJA‐responsive gene, followed by *DhWOX6* and *DhWOX7*, suggesting that they participate in resistance during early hormone induction. *AtWOX13* is a gene with high homology on the same branch as *DhWOX2* and is expressed mainly in meristematic tissues, including the replum. The *wox13* mutations reduce the replum size and enhance the phenotypes of mutants affected by the replum identity gene (Romera‐Branchat et al. 2013). *AtWOX13* functions as a key regulator of callus formation and organ adhesion in *Arabidopsis*. *WOX13* expression was rapidly induced upon wounding and was partly dependent on the activity of *wound‐induced dedifferentiation 1*. The *wox13* mutant is defective in establishing organ reconnection upon grafting, implying that *WOX13* is crucial for tissue repair (Ikeuchi et al. 2022). Recent studies confirmed that *WOX13* plays a key role in determining the cellular identity of callus cell populations. *WOX13* negatively regulates SAM formation from calli in *Arabidopsis* via transcriptional repression of *WUS* and other SAM regulators (Ogura et al. 2023). *DhWOX6* and *DhWOX7* are major genes that respond to MeJA stress, and their expression levels increase rapidly within 1 h. Phylogenetic analysis revealed that they share high homology with *AtWOX3* and *AtWOX8*. AtWOX3/PRS1 is involved in lateral organ development by recruiting founder cells to form lateral domains (Shimizu et al. 2009). *SlWUS* was mainly expressed in shoot apices and flower buds, of which *SlWOX3a* and *SlWOX3b* were mainly expressed in young leaves, shoot apices, and flower buds, and these two WOX genes were also significantly induced by MeJA (Lian et al. 2014; Li et al. 2021). Expression of *DhWOX8* and *DhWOX9* was relatively delayed in the presence of MeJA, peaking at 4 h. We observed that they were located in the WUS and intermediate clades, respectively. DhWOX8 is highly homologous to AtWOX2, whereas DhWOX9 belongs to the same branch as AtWOX11/12, suggesting that they may have different regulatory functions. *AtWOX2* is expressed in zygotes and early embryogenesis and functions in correcting the apical domain development of embryos (Haecker et al. 2004). *AtWOX2* triggers the expression of *PINFORMED1*, an auxin transporter that localizes auxin to the cotyledonary tips of early embryos and root poles (Breuninger et al. 2008). However, AtWOX11 plays a key role in the differentiation of vascular cambium into new lateral root founder cells. AtWOX11 is strongly induced and expressed during *de novo* root organogenesis, which is the same as its homologous AtWOX12 (Baesso et al. 2018).

## 5 Conclusion

In this study, nine *WOX* genes were identified in the *D. huoshanense* genome, with *DhWOX* genes exhibiting varying intron numbers, from one to three. Cis‐regulatory elements in the promoter regions of the *DhWOX* gene were found to respond to light, developmental stages, hormone signaling, indicating their potential in helping *DhWOX* plants deal with abiotic stresses. Whole‐genome duplication analysis revealed that the *DhWOX* gene did not originate from WGD or segmental duplication, but likely originate from proximal and dispersed duplication. Structural variation analysis showed that the genomes of *D. huoshanense* and *D. nobile* showed significant inversions and translocations, the most obvious of which was an inversion on chromosome 6. Transcriptome analysis revealed that *DhWOX*6, *DhWOX7*, and *DhWOX9* may function based on the specific MeJA concentrations and optimal timeframes. In addition, *DhWOX1* and *DhWOX3* showed no detectable expression during this time interval, indicating a lack of response to MeJA under the applied conditions. Further studies, including gene cloning and functional analyses, are essential to validate the significance of these genes in various physiological and biological processes.

## Author Contributions

**Jing Wang:** funding acquisition (equal), investigation (equal), validation (equal), visualization (equal), writing – review and editing (equal). **Yingyu Zhang:** formal analysis (equal), investigation (equal), software (equal), validation (equal), writing – original draft (equal), writing – review and editing (equal). **Yanshuang Ren:** formal analysis (equal), software (equal), writing – original draft (equal). **Muhammad Aamir Manzoor:** methodology (equal), supervision (equal), writing – review and editing (equal). **Shanyong Yi:** funding acquisition (equal), writing – review and editing (equal). **Cheng Song:** conceptualization (equal), data curation (equal), funding acquisition (equal), project administration (equal), resources (equal), supervision (equal), writing – original draft (equal), writing – review and editing (equal).

## Supporting information


Table S1.

Table S2.

Table S3.

## Data Availability

The datasets used in this study are included in the manuscript and additional files. The genome sequence of *D. huoshanense* was downloaded from the NCBI database under the accession code PRJNA597621. The raw sequence data of the transcriptome used in this study were obtained from the NGDC GSA database with the accession code CRA006607.
